# Functional Characterization of the Origin of Replication of pRN1 from *Sulfolobus islandicus* REN1H1

**DOI:** 10.1371/journal.pone.0084664

**Published:** 2013-12-20

**Authors:** Chijioke J. Joshua, Luis D. Perez, Jay D. Keasling

**Affiliations:** 1 Department of Plant and Microbial Biology, University of California, Berkeley, Berkeley, California, United States of America; 2 Department of Chemical & Biomolecular Engineering, University of California, Berkeley, Berkeley, California, United States of America; 3 Department of Molecular and Cell Biology, University of California, Berkeley, Berkeley, California, United States of America; 4 Physical Biosciences Division, Lawrence Berkeley National Laboratory, Berkeley, California, United States of America; 5 Joint BioEnergy Institute, Emeryville, California, United States of America; 6 Department of Bioengineering, University of California, Berkeley, Berkeley, California, United States of America; University of Illinois, Urbana-Champaign, United States of America

## Abstract

Plasmid pRN1 from *Sulfolobus islandicus REN1H1* is believed to replicate by a rolling circle mechanism but its origin and mechanism of replication are not well understood. We sought to create minimal expression vectors based on pRN1 that would be useful for heterologous gene expression in *S. acidocaldarius*, and in the process improve our understanding of the mechanism of replication. We constructed and transformed shuttle vectors that harbored different contiguous stretches of DNA from pRN1 into *S. acidocaldarius E4-39*, a uracil auxotroph. A 232-bp region 3’ of *orf904* was found to be critical for pRN1 replication and is therefore proposed to be the putative origin of replication. This 232-bp region contains a 100-bp stem-loop structure believed to be the double-strand origin of replication. The loop of the 100-bp structure contains a GTG tri-nucleotide motif, a feature that was previously reported to be important for the primase activity of Orf904. This putative origin and the associated *orf56* and *orf904* were identified as the minimal replicon of pRN1 because transformants of plasmids lacking any of these three features were not recovered. Plasmids lacking *orf904* and *orf56* but harboring the putative origin were transformable when *orf904* and *orf56* were provided *in-trans*; a 75-bp region 5’ of the *orf904* start codon was found to be essential for this complementation. Detailed knowledge of the pRN1 origin of replication will broaden the application of the plasmid as a genetic tool for *Sulfolobus* species.

## Introduction

*Sulfolobus acidocaldarius* is a hyperthermophilic, aerobic crenarchaea that grows optimally at 75°C and pH 2 – 4 [1]. *S. acidocaldarius* is capable of utilizing mixtures of sugars such as glucose and xylose simultaneously as sources of carbon and energy without an apparent diauxie [2-5], making it an ideal organism for production of cellulosic biofuels. The genomes of most *Sulfolobus* species, with the exception of *S. acidocaldarius*, are fairly unstable due the presence of large numbers of active insertion elements [1,6]. Due to its genome stability, *S. acidocaldarius* is a great model for understanding the physiology of archaea [1,6]. Genetic tools for the study of *Sulfolobus* species have improved greatly, especially within the past five years [7-12]. Although it is possible to integrate into the chromosome of *Sulfolobus* species [9,10,12], very few plasmid systems have been shown to reproducibly replicate in *Sulfolobus* species. Development of more effective and reproducible plasmids would greatly enhance the application of these organisms as microbial platforms for biotechnology. Advancement of genetic tools for *Sulfolobus* species is also adversely impacted by the absence of effective selectable markers. The commonly used selection strategies are based on uracil auxotrophy or lactose selection [7,13-16]; the use of antibiotics for *Sulfolobus* has been reported [17], but this strategy has not been very successful because of low susceptibility of archaea to antibiotics [18,19]. 

Shuttle vectors are crucial for gene expression in non-model organisms such as *S. acidocaldarius*. Shuttle vectors based on pRN1 are among the most successful vehicles for heterologous gene expression in *Sulfolobus* species [7,20]. The small cryptic plasmid (pRN1) from *Sulfolobus islandicus REN1H1* is a self-replicating, multi-copy plasmid [21]. The plasmid belongs to the pRN family that replicate via the rolling circle mechanism; however, its origin of replication has not been fully elucidated [7,22-28]. 

Rolling circle plasmids generally contain two origins of replication: a double-strand origin (dso), which initiates leading strand replication, and a single-strand origin (sso), which initiates replication of the lagging strand. The double-strand origin contains inverted repeats that form a stem-loop or ‘cruciform’ structure [28,29]. A replication protein nicks a site on the outer DNA strand within the loop of the IRII arm of the cruciform structure [28-32]. The nick exposes the 3’ OH group, which serves a primer for the synthesis of a new DNA strand (leading strand) around the inner strand by a DNA polymerase, displacing the outer strand (lagging strand) in the process. Replication of the lagging strand is initiated at the single-strand origin (sso) which usually located at the 5’ of the *dso* and exposed after the synthesis of the leading strand [28,29]. A detailed description of this mechanism has been reviewed by Khan [29]. 

Shuttle vectors based on pRN1 and the closely related pRN2 [7,8] are generally larger than 8 kb [7,20]. The development of smaller pRN1-based plasmids would simplify expression of heterologous genes in *S. acidocaldarius*. Knowing which components of the plasmid are essential for replication would facilitate minimizing the plasmid’s size. There are six genes on pRN1, two of which, *orf56* and *orf904*, are co-transcribed and highly conserved within the pRN family of plasmids, and have also been reported to be essential for plasmid replication [7,22,25,33]. A third gene (*orf80*) is less conserved, while the other genes are not conserved within the pRN family. The protein encoded by *orf56* (CopG) is proposed to regulate plasmid copy number and its own level by binding to its own promoter [25,34]. Orf904 is a large, multi-functional, replication protein with helicase, primase, and polymerase domains [25]. Orf904 is believed to initiate replication by binding to the plasmid’s double-strand origin of replication; its primase domain was also reported to require a tri-nucleotide (GTG) motif for activity [35]. Comparative analysis of pRN1 and other members of the pRN family of plasmids suggested that the single-strand origin (sso) and double-strand origin (dso) of replication of pRN1 are located 3’ of *orf904*, but the exact origin of replication has not been confirmed experimentally [23,25,27].

In this study, we characterized the role of a 232-bp region downstream of *orf904* as the putative origin of replication of pRN1 using a number of shuttle vectors harboring various lengths of pRN1. Our results indicate that a 100-bp stem loop structure 3’ of *orf904* is critical for plasmid replication, as deletions within this structure were not tolerated. The minimum replication unit of pRN1 was confirmed to consist of *orf56*, *orf904*, and the region harboring the stem-loop structure. 

## Materials and Methods

### Strains and growth media

*S. acidocaldarius E4-39* is a spontaneous uracil auxotroph (Figure S1) that was isolated from *S. acidocaldarius 639* from DSMZ (Braunschweig, Germany). The strains were cultivated aerobically on ATCC #1723 (YT) medium. The organism was generally grown in liquid medium at 75°C on a rotary water-bath shaker at 200 rpm and at 70°C on solid medium that was composed of YT or T (YT without yeast extract) medium solidified with 0.8% gellan gum (Spectrum and Sigma). The pH of each growth medium was adjusted to 2.5 with 5 M H_2_SO_4_, and each medium was filter sterilized using 0.2-µm pore size membrane filters. Chemicals used in this study were obtained from Sigma, Spectrum, and Fisher scientific. 

### Construction of shuttle vectors

**Figure 1:**
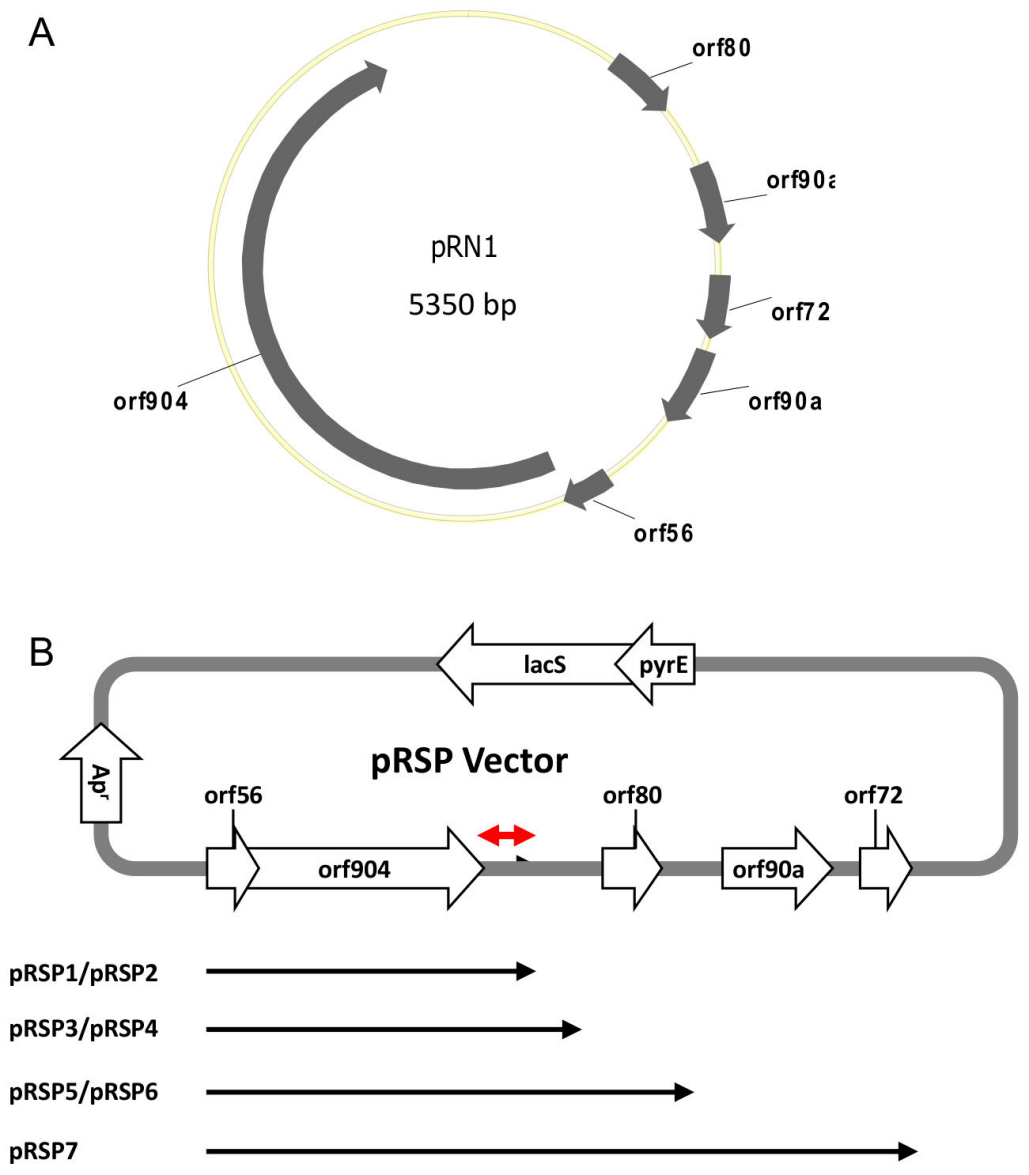
Construction of pRN1-based shuttle vectors. (A) Map of pRN1 showing native genes. (B) Map of pRN1-based shuttle vector(s) showing the 232-bp putative pRN1 origin of replication (red double arrow). Each vector harbors the pUC19 Ap^r^ gene and origin (red box), *pyrE* with or without *lacS* and a segment of pRN1 highlighted by black arrows. The *lacS* gene was deleted from pRSP1, 3, and 5 to generate pRSP2, 4, and 6.

Seven shuttle vectors (pRSP1 – pRSP7) harboring four lengths of pRN1, pUC19 and a cassette (*pyrE-lacS*) containing *pyrE* and *lacS* genes from *S. solfataricus* were assembled by a PCR-based cloning method to verify the minimal sequence of pRN1 that is critical for plasmid replication (Figure 1). Three of the shuttle vectors pRSP2, pRSP4 and pRSP6 were constructed by eliminating *lacS* from pRSP1, pRSP3 and pRSP5, respectively, by PCR. Each vector contained a 232-bp sequence located 3’ of *orf904* that we identified as the putative origin of the plasmid by inspection of un-annotated pRN1 sequence based on A-T abundance. The first set of plasmids (pRSP1, pRSP3, pRSP5 and pRSP7) was constructed by assembling three PCR-amplified DNA fragments: the 1750-bp pUC19 fragment, 2239-bp *pyrE*-*lacS* cassette, and a pRN1 fragment (Figure 1 and Table 1). The pUC19 fragment (nt. 800 - 2550) was amplified with primers P/N128 and P/N129 and consists of *bla* (Amp^R^) and the pMB1 origin of replication (Table S1). The *pyrE*-*lacS* cassette was amplified from pGlcSTUV_ko (unpublished plasmid, Figure S2) with primers P/N117 and P/N120 and includes the contiguous 80-bp 3’ of the *lacS* stop codon, which is assumed to contain the native terminator sequence (Figure 1). Translation of *lacS* was placed under the control of putative RBS of *pyrF* located at the 3’-end of *pyrE* and was kept in frame by the addition of an A-T base pair between the stop codon of *pyrE* and the start of *lacS*. The *pyrE-lacS* operon was transcribed by the promoter of the beta subunit (*thsB*) of *S. solfataricus* chaperonin (Sso0282). . The four pRN1 fragments were amplified from shuttle vector pC [7] using a forward primer (P/N130) and four reverse primers (P/N131 – 134) described in Table S1. 

**Table 1:** Description of plasmids and strains.

**Plasmid/Strains**			Description of important features*	**Source/ Reference**
**Plasmids**				
	pUC19		pMB1 origin, AmpR, (2.7 kb)	
	Pc		pRN1 (entire plasmid); pBluescript; *pyrEF* (9.0 kb)	3
	pRSP1		pRN1 (*orfs56*/*904*), AmpR, *pyrE* and *lacS* (7.2 kb)	This study
	pRSP1-NO		4681 - 4912 deleted from pRSP1 (6.9 bp)	This study
	pRSP1a		4853 - 4912 deleted from pRSP1 (7.1 kb)	This study
	pRSP1b		4793 - 4852 deleted from pRSP1 (7.1 kb)	This study
	pRSP1c		4733 - 4792 deleted from pRSP1 (7.1 kb))	This study
	pRSP1d		4681 - 4732 deleted from pRSP1 (7.1 kb)kb)	This study
	pRSP1e		4844 - 4855 deleted from pRSP1 (7.2 kb))	This study
	pRSP1-L1		4793 - 4812 deleted from pRSP1 (7.1 kb)	This study
	pRSP1-L2		4773 - 4792 deleted from pRSP1 (7.1 kb)	This study
	pRSP1-L3		4755 - 4772 deleted from pRSP1 (7.1 kb)	This study
	pRSP1-L4		4755 - 4812 deleted from pRSP1 (7.1 kb)	This study
	pRSP1-G1		4773 - 4775 deleted from pRSP1 (7.2 kb)	This study
	pRSP1-G2		4776 - 4778 deleted from pRSP1 (7.2 kb)	This study
	pRSP1-G3		4779 - 4781 deleted from pRSP1 (7.2 kb)	This study
	pRSP1-G4		4782 - 4784 deleted from pRSP1 (7.2 kb)	This study
	pRSP1-G5		4785 - 4787 deleted from pRSP1 (7.2 kb)	This study
	pRSP1-G6		4788 - 4790 deleted from pRSP1 (7.2 kb)	This study
	pRSP1-G7		4791 - 4793 deleted from pRSP1 (7.2 kb)	This study
	pRSP1-G8		4794 - 4796 deleted from pRSP1 (7.2 kb)	This study
	pRSP1-A1		A→G substitution at position 4784	This study
	pRSP1-A2		A→G substitution at position 4786	This study
	pRSP1-A3		A→G substitution at position 4787	This study
	pRSP1-A7		A→G substitution at position 4794	This study
	pRSP1-CL		pRSP1with XhoI-AatII-NruI-PacI-PstI-PvuII-NsiI site (7.2 kb)	This study
	pRSP2		*lacS* deleted from pRSP1(5.7 kb)	This study
	pRSP2-CL		pRSP2 with HindIII-XhoI-AatII-NruI-PacI-PstI- PvuII-NsiI sites (5.7 kb)	This study
	pRSP3		pRN1 (*orfs56*/*904*), AmpR, *pyrE* and *lacS* (7.6 kb)	This study
	pRSP3-NO		4707 - 4912 deleted from pRSP3 (7.4 kb)	This study
	pRSP4		*lacS* deleted from pRSP3 (6.1 kb)	This study
	pRSP5		pRN1 (*orfs56*/*904/80*), AmpR, *pyrE* and *lacS* (8.1 kb)	This study
	pRSP6		*lacS* deleted from pRSP5 (6.6 kb)	This study
	pRSP7		pRN1 (*orfs56*/*904/80/90a/72*), AmpR, *pyrE* and *lacS* (8.8 kb)	This study
	pRSP9		*orf904* (1986 - 4680) deleted from pRSP1(4.5 kb)	This study
	pRSP10		*orf56* (1815 - 1965) deleted from pRSP1 (7.0 kb)	This study
	pRSP10b		Portion of *orf56* (1815 - 1890) deleted from pRSP1 (7.1 kb)	This study
	pGlcSTUV_ko		Source of *pyrE-lacS* cassette (6.9 kb)	Unpublished
**Strains**				
		GX3	*S. acidocaldarius* (DSMZ 639) utilizes glucose and galactose)	14
		E4-39	Uracil auxotroph with 17 bp duplication in pyre	This study
		E4-RSP1	Strain E4-39 harboring pRSP1	This study
		E4-RSP1-CL	Strain E4-39 harboring pRSP1-CL	This study
		E4-RSP2	Strain E4-39 harboring pRSP2	This study
		E4-RSP2-CL	Strain E4-39 harboring pRSP2-CL	This study
		E4-RSP3	Strain E4-39 harboring pRSP3	This study
		E4-RSP4	Strain E4-39 harboring pRSP4	This study
		E4-RSP5	Strain E4-39 harboring pRSP5	This study
		E4-RSP6	Strain E4-39 harboring pRSP6	This study
		E4-RSP7	Strain E4-39 harboring pRSP7	This study

^*^ *pyrE, pyrF* and *lacS* are from *S. solfataricus P2*; AmpR gene obtained from pUC19

We assembled pRSP1, pRSP3, pRSP5, and pRSP7 using a modified SLIC cloning strategy [36,37] by combining the pUC19 and *pyrE-lacS* fragments with one of the four pRN1 fragments in a 30-µl chew-back and annealing (CBA) reaction with all fragments at approximately the same molarity using 50 - 100 ng of the pUC19 fragment as reference. The CBA reaction contained 1X T4 ligase buffer (Fermentas), 0.1 mg/mL BSA, 0.75 µL T4 polymerase (Fermentas) and water. The reaction was carried out in a thermocycler at 37°C for 7 min, then at 75°C for 20 min, and cooled to 60°C at 0.1°C /s. The reaction was held at 60°C for 30 min, and then cooled to 4°C at 0.1°C /s [37]. A fill-in/ligation (PL) reaction was carried out by adding 0.2 µl T4 polymerase, 0.2 µl 10 mM dNTP’s, 0.2 µl 50 mM ATP, and 0.5 µl T4 ligase to 10 µl of the CBA reaction, incubating at 37°C for 30 min, and then inactivating the enzyme at 75°C for 5 min. Samples from each cloning reaction (CBA and PL) were transformed into *E. coli* DH10B and TOP10 and inoculated onto LB plates containing 100 µg/ml carbenicillin. Constructed vectors were extracted and verified by sequencing the entire plasmid. We subsequently constructed pRSP2, pRSP4 and pRSP6 by removing *lacS* from pRSP1, pRSP3, and pRSP5, respectively, using P/N135 and P/N136 as forward and reverse primers, respectively (Table S1). A 30-bp adaptor that is complementary to the 3’-end of *pyrE* was added to the 5’-end of primer P/N135 to ensure amplification of circular plasmids. Details of the primers used in amplifying these fragments can be found in Table S1. 

### Mapping the putative origin of replication of pRN1

**Figure 2:**
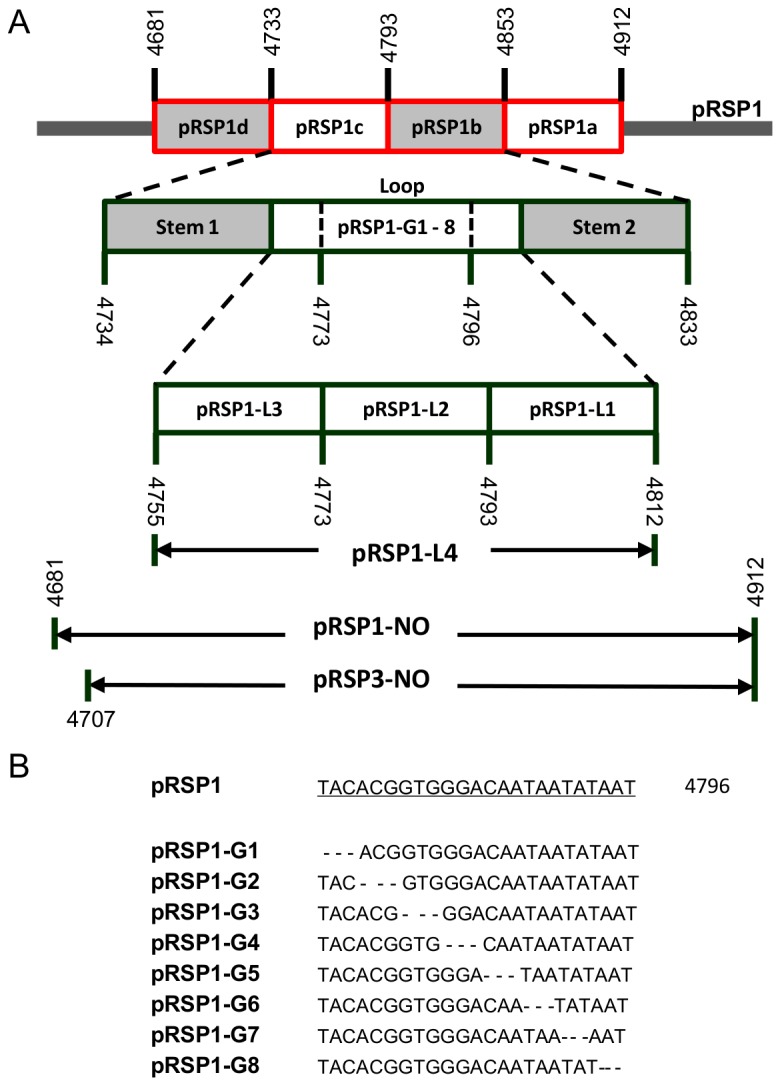
Strategy of mapping pRN1 putative origin of replication (double red arrow in Figure 1). (A) Various deletions were made within the ‘putative ori’ in pRSP1 to generate plasmids highlighted in each rectangular box to determine the role of the deleted regions in pRN1 replication. We constructed pRSP1-NO and pRSP3-NO by deleting 232-bp and 207-bp, respectively, from the putative origin. (B) Three-nucleotide deletion mutants of pRSP1 were constructed to map the loop of stem-loop structure within pRN1 origin. Each construct was transformed into *S. acidocaldarius* and scored for production of LacS expressing transformants.

The 232-bp putative origin of replication was functionally mapped by partial or complete deletion of this region from pRSP1 or pRSP3 (Figure 2 and Table 1). The resulting plasmids were methylated, transformed into *S. acidocaldarius E4-39*, and scored for their ability to generate transformants. First, we divided the 232-bp region into four segments of 52- or 60-bp and deleted each segment from pRSP1 to generate four plasmids (pRSP1a – d) (Table 1 and Figure 2A). We also generated a fifth plasmid (pRSP1e) by deleting a 12-bp sequence (4844 - 4855) that includes a GTG tri-nucleotide motif from pRSP1 (Table 1) to determine its role in plasmid replication. Next, we mapped the 58-bp loop (4755 - 4812) of a putative stem-loop (SL) secondary structure (4734 - 4833) within the 232-bp putative origin in pRN1 to determine if it is critical for plasmid replication. The loop (L) was mapped by deleting 18- or 58-bp segments from this loop in pRSP1, generating four plasmids (pRSP1-L1 – pRSP1-L4) (Table 1 and Figure 2A). We generated a third set of plasmids (pRSP1-G1 – 8) making 3-bp deletions from pRSP1 (Figure 2) to further analyze the 24-bp stretch of DNA (4773 - 4796) within the 58-bp loop that contained a putative GTG motif reported to be important for primase activity of Orf904 [35]. The entire 232-bp putative origin (4681 - 4912) was deleted from pRSP1 to generate pRSP1-NO to test for loss of function; a similar construct (pRSP3-NO) was generated by deleting 207 of the 232-bp (4707 - 4912) from pRSP3. With the exception of pRSP1b and pRSP1d, each plasmid described above was amplified directly from pRSP1 as a linear, blunt-end, PCR fragment lacking the targeted region. Approximately 0.2 - 0.3 pmol of the 5’-terminus of each linear PCR amplicon was phosphorylated at 37°C for 20 min with T4 polymerase kinase (PNK) and ligated overnight at ~22°C using T4 ligase as described by the manufacturer (Fermentas). The plasmids pRSP1b and pRSP1d were amplified as circular plasmids using primers with adaptors with overlapping sequence (Table S1). Each construct was transformed into either *E. coli* DH10B or TOP10, and deletions in the generated vectors were confirmed by sequencing the surrounding regions. These were subsequently methylated and transformed into *S. acidocaldarius* and screened for blue colonies expressing LacS. The primers used in the construction of these plasmids are listed in Table S1. 

### Methylation and transformation of *S. acidocaldarius*

Each assembled shuttle vector was transformed into *E. coli* 1821.pM.EsaBC4I (New England Biolabs) for methylation to protect it against SuaI, a restriction endo-nuclease present in *S. acidocaldarius* [38]. Approximately 1 - 2 µl of each methylated construct was transformed into 80 µl of electro-competent, uracil-auxotrophic *S. acidocaldarius* E4-39 cell suspension (OD_600_ = 5 - 10) that was prepared following the “G” procedure described by Kurosawa and Grogan [39]. Prior to the addition of plasmid DNA, the competent cell suspension was diluted with approximately 5 parts of ice-cold 1% sucrose solution. The mixture was transferred into a 1-mm cuvette; electroporated at 1.25 KV, 50 µF, and 750 Ω; and immediately recovered with 80 µl of ice-cold 2X recovery buffer (1% sucrose, 20 mM β-alanine/1.5 mM malate buffer [pH = 4.5] and 10 mM MgSO_4_). A negative control reaction (without plasmid DNA) was added to every transformation experiment. The recovered cells were incubated at 70°C for 30 min, cooled on ice for 2 - 5 min, and spread on YT or T plates. The plates were incubated at 70°C in humidified Sterilite™ plastic containers for 5 - 7 days. After incubation, the plates were sprayed with 5 mg/ml X-gal (5-romo-4-chloro-3-indolyl-b-D-galactoside) in dimethyl formamide (DMF) to qualitatively screen for LacS activity. Blue colonies were scored as positive selected for further characterization. Small to medium-sized (white) colonies were selected as positive transformants from the transformation reaction with plasmid lacking *lacS* (pRSP2, pRSP4 and pRSP6) upon comparing these plates to the negative control plate. Most transformation experiments were repeated at least two times to verify the obtained result. To recover plasmid DNA, each selected transformant was grown in 50 ml of YT medium (OD_600_ ≈ 1.0). The cells were pelleted and re-suspended in 500 μl of P1 buffer supplemented with 15 mg/ml lysozyme to enhance lysis. The re-suspended cells were incubated at 37°C for 10 min, and then treated with 500 μl of buffer P2 and 700 μl of buffer N3 following the protocol described by the manufacturer. The recovered plasmid was re-transformed into *E. coli*. Plasmids recovered from *S. acidocaldarius E4-39* and re-transformed into *E. coli* were treated with EcoRI to determine the restriction digestion fingerprint.

### Plasmid complementation

We constructed pRSP9 and pRSP10 by making in-frame deletions of *orf904* and *orf56*, respectively, from pRSP1 (Table 1). A third plasmid, pRSP10b, was constructed by retaining 75-bp sequence upstream of the *orf904* start codon. The plasmids were constructed by PCR amplification of the entire pRSP1 without the gene segment targeted for deletion using primers described in Table S1 and the protocol described above. Each construct was methylated and transformed into *S. acidocaldarius E4-39*; pRSP9 was paired with pRSP10 or pRSP10b and transformed into the host to test for gene complementation. 

### Bioinformatics analyses

The plasmids used in this study were designed and analyzed using Invitrogen’s Vector NTI software. DNA sequences were aligned and analyzed using BLAST and ClustalW2 informatics tools which can be accessed via http://www.ncbi.nlm.nih.gov and http://www.ebi.ac.uk/Tools/clustalw2/index.html. 

### Nucleotide sequence access

The nucleotide sequences of the plasmids used in this study have been deposited in the Joint BioEnergy Institute (JBEI) public part registry and can be accessed via this link: https://public-registry.jbei.org/ #page=collections;id=102.

## Results

### The 232-bp putative ‘origin’ of pRN1 is critical for plasmid function

**Figure 3:**
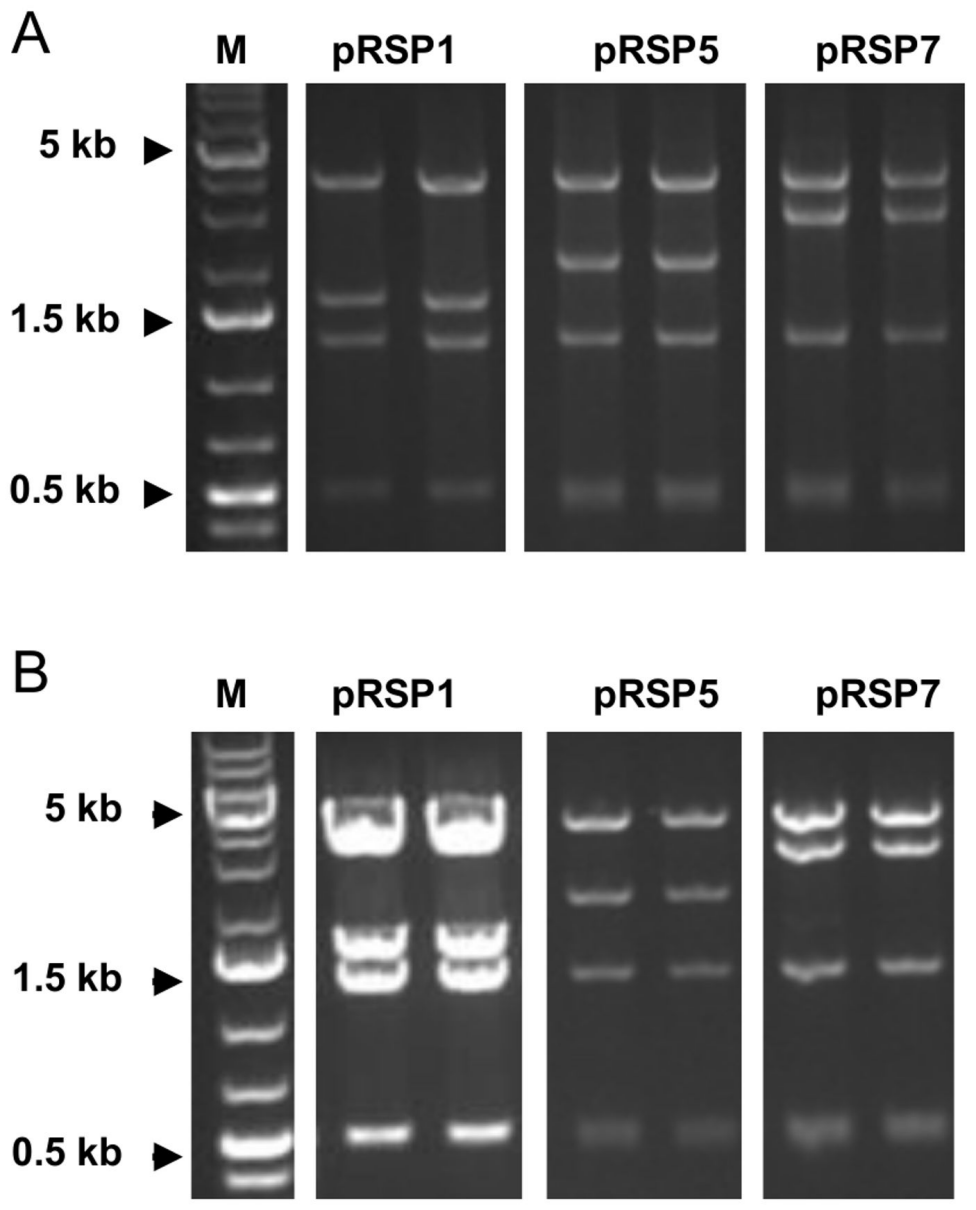
Extraction plasmids from *S. acidocaldarius* transformants and re-transformation into *E. coli*. (A) pRN1-based shuttle vectors were recovered from *S. acidocaldarius* transformants and re-transformed into *E. coli* to verify that the plasmids were self-replicating. The identity of the plasmids was confirmed by their EcoRI digestion finger-print. (A) EcoRI digestion finger-print of pRSP1, 5 and 7 recovered from (A) *S. acidocaldarius* *E4-39* transformants and (B) *E. coli* re-transformed with plasmid DNA from the S. *acidocaldarius* transformants analyzed in (A).

The origin of replication is critical for the function and replication of a plasmid in a suitable host. We evaluated the impact of a 232-bp DNA sequence on the ability of pRN1 to replicate and yield transformants. *Sulfolobus* species have been successfully transformed with pRN1-based plasmids but the origin of replication of this plasmid has not been fully characterized. The 232-bp stretch of DNA (5031 - 5262) was identified by visual inspection of the pRN1 sequence and was found to be located at the 3’ of *orf904* in agreement with previous report [25]. We evaluated the function of the 232-bp stretch of DNA by constructing shuttle vectors (pRSP1 – pRSP7) bearing various lengths of pRN1 (Figure 1B and Table 1) and transforming the plasmids into *S. acidocaldarius E4-39*, a uracil auxotroph. All shuttle vectors yielded uracil prototrophic colonies of *S. acidocaldarius E4-39*, suggesting that each harbored the origin of replication (Table 2 and Figure S3). Colonies from pRSP1, 3, 5, and 7 transformations stained blue on X-gal plates due to β-glycosidase activity encoded by the S. *solfataricus lacS* present on these plasmids, whereas colonies from pRSP2, 4, and 6 remained white with X-gal exposure due to the absence of *lacS* (Figure S3). The transformations were repeated to confirm the results. The sequence of each plasmid was confirmed by sequencing the entire plasmid prior to transformation into *S. acidocaldarius E4-39*. We also verified that each plasmid was self-replicating and not integrated into the host genome by recovering the plasmids from each transformants and re-transforming the recovered plasmid into *E. coli*. The EcoRI digestion fingerprint of plasmids recovered from *S. acidocaldarius E4-39* was identical to those recovered from re-transformed *E. coli* indicating that the plasmids were self-replicating (Figure 3). 

**Table 2:** Transformation efficiency of constructed shuttle vectors into *S. acidocaldarius* *E4-39*.

**Plasmid(s**)	**pRN1 fragment**	**pRN1 fragment location on shuttle vector**	**Length of pRN1 fragment**	**Transformation yield (cfu/ng**)***^a^*,*^b^***
pRSP1	2101 - 5270	1751 - 4920	3170	7.7 × 10^3^
pRSP2	2101 - 5270	1751 - 4920	3170	4.9× 10^3^ *
pRSP3	2101 - 314	1751 - 5314	3564	3.3 × 10^3^
pRSP4	2101 - 314	1751 - 5314	3564	4.0 × 10^3^ *
pRSP5	2101 - 814	1751 - 5814	4064	1.3 × 10^3^
pRSP6	2101 - 814	1751 - 5814	4064	2.2 × 10^4^ *
pRSP7	2101 - 1614	1751 - 6614	4864	3.5 × 10^2^

^a^ Most transformation experiments were carried out at least two time and efficiency varied with experiments

^b^ Negative control (without plasmid DNA) was always included with each set of experiments

^*^ Transformants were classified as small to medium colonies

The transformation results indicate that pRSP1 and pRSP2, which harbored *orf56, orf904*, and the 232-bp sequence (Table 2 and Figure 1B), constitute the minimum replication unit of pRN1, in agreement with a previous report [25]. Replication of pRSP1 and pRSP2 suggests that the 232-bp sequence harbors the putative origin of replication. To further confirm the function of the 232-bp sequence, we deleted the feature from pRSP1 and pRSP3. The resulting plasmids (pRSP1-NO and pRSP3-NO) failed to yield transformants (Table 3). The experiment was repeated at least two times, and on every occasion transformants were recovered with pRSP1 and pRSP3, while pRSP1-NO and pRSP3-NO failed to yield transformants. The critical role of the 232-bp putative origin of replication in the propagation of pRN1 was further highlighted by the inability of deletion mutants (pRSP1-NO and pRSP3-NO) lacking this region to yield transformants, while the parent plasmids, pRSP1 and pRSP3, respectively, always yielded transformants (Table 3). The result was highly reproducible and was confirmed on at least three separate occasions. The absence of transformants from pRSP1-NO and pRSP3-NO suggests that *orf56* and *orf904* or the additional pRN1 sequence in pRSP3 (4913 - 5314) are insufficient for pRN1 replication, thus highlighting the importance of the 232-bp sequence. 

**Table 3:** Effect of deletion within putative origin of pRSP1 and pRSP3 on transformation efficiency.

**Plasmid(s**)	**Regions deleted**	**Length**	**Affected feature**	**Transformation yield (cfu/ng**)***^a^*,*^b^***
pRSP1	Nil	Nil	Putative origin in pRSP1	4.8 - 8.4 × 10^3^
pRSP1a	4853 - 4912	60		4.8 – 6.6 × 10^3c^
pRSP1b	4793 - 4852	60		Nil
pRSP1c	4733 - 4792	60		Nil
pRSP1d	4681 - 4732	52		1.5 × 10^3^ – 1.0 × 10^4^c
pRSP1e	4844 - 4855	12		5.2 × 10^3^ – 1.0 × 10^4^
pRSP1-NO	4681 - 4912	232		Nil
pRSP1-L1	4793 - 4812	20	100-bp stem-loop	Nil
pRSP1-L2	4773 - 4792	20		Nil
pRSP1-L3	4755 - 4772	20		Nil
pRSP1-L4	4755 - 4812	20		Nil
pRSP1-G1	4773 - 4775	3	Loop of 100-bp stem-loop	Nil
pRSP1-G2	4776 - 4778	3		Nil
pRSP1-G3	4779 - 4781	3		Nil
pRSP1-G4	4782 - 4784	3		Nil
pRSP1-G5	4785 - 4787	3		Nil
pRSP1-G6	4788 - 4790	3		Nil
pRSP1-G7	4791 - 4793	3		Nil
pRSP1-G8	4794 - 4796	3		Nil
pRSP3	Nil	Nil	Putative origin in pRSP3	2.2 - 3.2 × 10^3^
pRSP3-NO	4707 - 4912	206		Nil

^a^ The transformation experiments were carried out at least three times and the efficiency varied with experiments

^b^ Negative control (without plasmid DNA) and positive control (pRSP1 or pRSP3) reactions were always included with each set of experiments

^c^ Colonies are very tiny compared to transformants from pRSP1

### The 232-bp region harbors a 100-bp putative replication initiation stem-loop

We mapped the 232-bp sequence by deleting the 52- or 60-bp segments from pRSP1 generating pRSP1a – pRSP1d (Table 2 and Figure 2). Two of the four plasmids (pRSP1b and pRSP1c) did not yield transformants when transformed into *S. acidocaldarius E4-39* on at least three different occasions (Table 3 and Figure S4). This result suggests that the regions deleted in pRSP1b and pRSP1c are critical for plasmid function. The other two plasmids (pRSP1a and pRSP1d) yielded transformants with a very tiny growth phenotype on solid medium (Table 3 and Figure S4). Transformants from pRSP1a grew poorly in liquid medium; hence we were not able to recover plasmid DNA, whereas pRSP1d transformants did not grow in liquid medium after several attempts. 

Comparative analysis of the 232-bp sequence with pRN2, pHEN7, and pDL10 revealed that the 232-bp sequence is fairly conserved within the pRN family (Figure S5). The alignment also showed that the 232-bp sequence in pRN1 is much closer in homology to that in pHEN7 than to that in pRN2 or pDL10. We identified a 12-bp sequence harboring a GTG motif that was conserved in all four plasmids (Figure S5) and deleted this motif from pRSP1 to generate pRSP1e (Table 1). It was previously reported that the primase activity of *orf904* requires a GTG motif [35]. The resulting plasmid (pRSP1e) replicated normally, indicating that this particular GTG motif was not essential for plasmid replication (Table 2).

**Figure 4:**
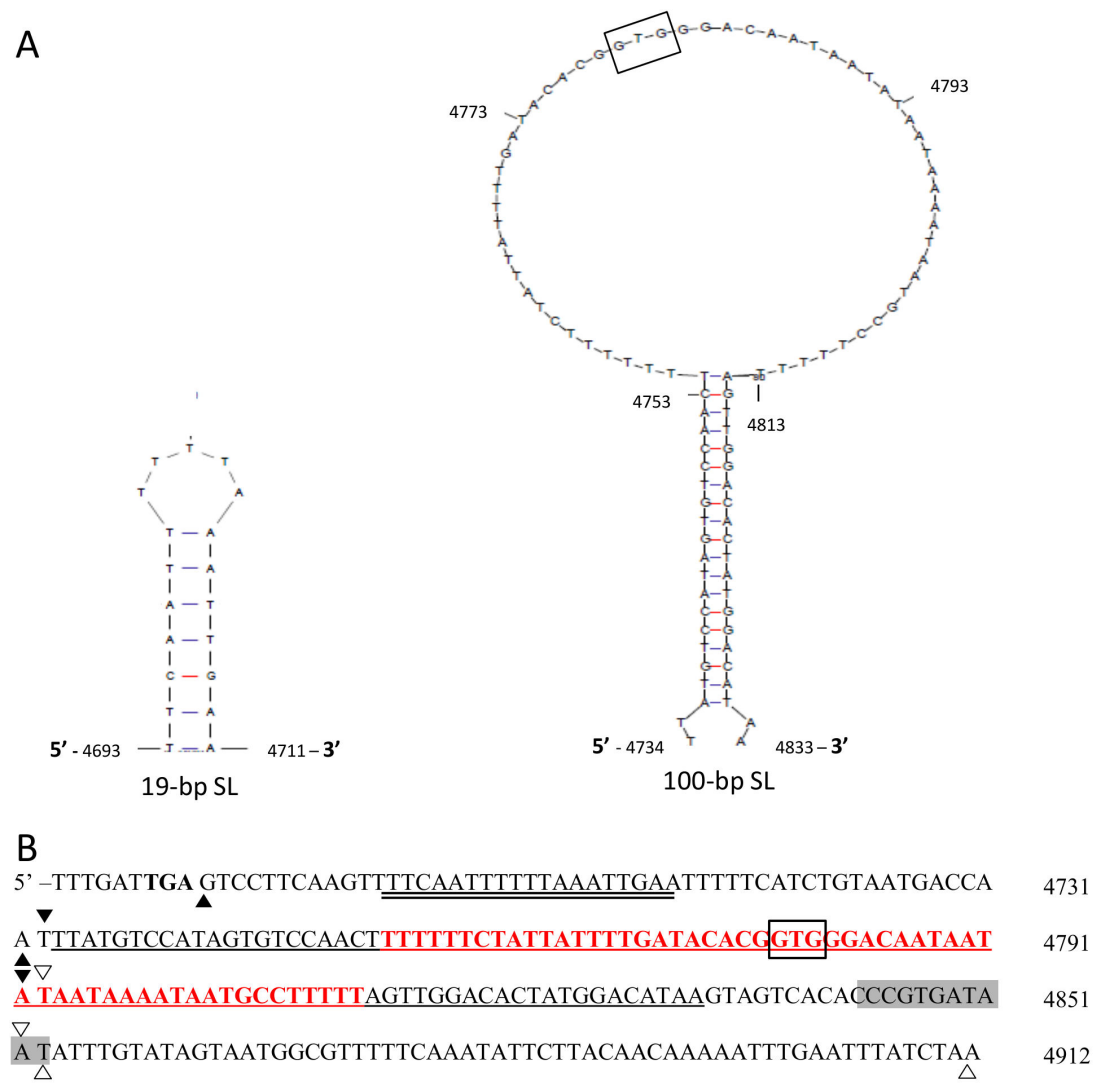
Stem-loop structures identified within the 232-bp pRN1 putative origin of replication. (A) The secondary structure of 232-bp putative origin of replication determined using DNA mFold, revealed the presence of 100-bp (4734 - 4833) and 19-bp (4693 - 4711) stem-loop (SL) structures at 70°C. (B) DNA sequence of the 232-bp putative origin of replication showing the 100-bp SL (single underline) and 19-bp SL (double underline) in pRN1 origin. Deletions made in pRSP1a (between ∆), pRSP1b (between∇), pRSP1c (between ▼) and pRSP1d (between ▲) are highlighted by the triangles, while the deletion in pRSP1e is shaded. The ‘GTG’ motif (box) and the loop of the 100-bp SL (red) are also highlighted.

 We determined the secondary structure of the 232-bp feature with mFold using 70°C, Na^+^ = 1.0 M and Mg^2+^ = 0.0 M as input parameters [40]. The analysis identified two stem-loop structures, a 19-bp structure at position 4693 - 4711 and a 100-bp structure at position 4734 - 4833 in pRSP1 (corresponding to 5043 - 5061 and 5084 - 5183 respectively, in pRN1) (Figure 4). A significant portion of the region encompassing the 100-bp stem-loop was deleted in pRSP1b and pRSP1c and could possibly explain the inability to recover transformants from cells transformed with these plasmids (Table 3). The 100-bp stem-loop structure is consistent with the structure of the critical double-strand origin of a rolling circle plasmid [28,29,41]. Lack of transformants from cells transformed with pRSP1b and pRSP1c suggests that part or the entire 100-bp stem-loop structure is critical for replication and likely site of replication initiation. 

The 19-bp stem-loop structure was part of the sequence deleted in pRSP1d that produced tiny *S. acidocaldarius E4-39* transformants that failed to grow in liquid medium. The role of this 19-bp structure is not clear, but based on its location at the 5’-end of the 100-bp stem-loop structure, we suspect that it might be part of the single-strand origin of replication [42]. It also possible to associate inability of pRSP1d transformants to grow in liquid medium to deletion of the 19-bp structure based on a previous report that deletion of the single-strand origin from a rolling circle plasmid results in plasmid instability [43]. The phenotype displayed by pRSP1d could not be attributed to deletion of the 19-bp stem-loop alone because the deleted sequence also contained two other potential small hairpin structures. Similar small potential hairpin structures are present in the region deleted in pRSP1a. Further work would be required to verify the actual function of the features deleted in pRSP1a and pRSP1d (Figure S6). 

### Loop of the 100-bp stem-loop structure is critical for replication

In pursuance of our proposal that the 100-bp stem-loop might be the double-strand origin (Figure 4), we deleted the 58-bp loop of the structure from pRSP1 to generate pRSP1-L4 (Figure 2). The loop of the 100-bp stem-loop structure could harbor the putative ‘nick site’ that initiates plasmid replication [28,41]. The 58-bp deletion mutant (pRSP1-L4) failed to yield *S. acidocaldarius* transformants (Table 3), suggesting that the deleted region was critical for plasmid replication. To further characterize this region, we divided the 58-bp loop into three segments, 18 or 20-bp in length, and deleted each segment from pRSP1 generating pRSP1-L1 – pRSP1-L3. Each plasmid (pRSP1-L1 – pRSP1-L3) reproducibly failed to produce *S. acidocaldarius E4-39* transformants after two attempts, compared to pRSP1, which consistently yielded transformants (Table 3). The inability to recover transformants with pRSP1-L1 – pRSP1-L4 supports the importance of the 58-bp loop for plasmid function, further highlighting the potential role of the 100-bp structure as the *cis*-acting double-strand origin of pRN1 (Figure 4). 

We further analyzed a GC-rich region in the middle of the 58-bp loop (4773 - 4786) by making 3-bp deletions from pRSP1 (Figure 2B), generating eight plasmids (pRSP1-G1 – pRSP1-G8). The G-C rich region contains a ‘GTG’ motif that was deleted in pRSP1-G3. Each plasmid was transformed into *S. acidocaldarius E4.39* along with the pRSP1 as a positive control. Only pRSP1 produced transformants; the other plasmids (pRSP1-G1 – pRSP1-G8) did not yield transformants after more than three attempts. The results from the 58-bp loop deletion mutants and those from pRSP1b and pRSP1c strongly highlight the critical nature of 100-bp stem-loop structure and its potential function as the putative double-strand origin of replication. We attempted to determine if the 58-bp is sequence specific by making a few single A → G base-pair substitutions 3’ of the ‘GTG’ motif in pRSP1, generating pRSP1-A1 (A4748G), pRSP1-A2 (A4786G), pRSP1-A3 (A4787G) and pRSP1-A7 (A4794G) and these plasmids yielded LacS-expressing transformants comparable to pRSP1. 

### The *trans*-acting Orf56 and Orf904 complementation

**Figure 5:**
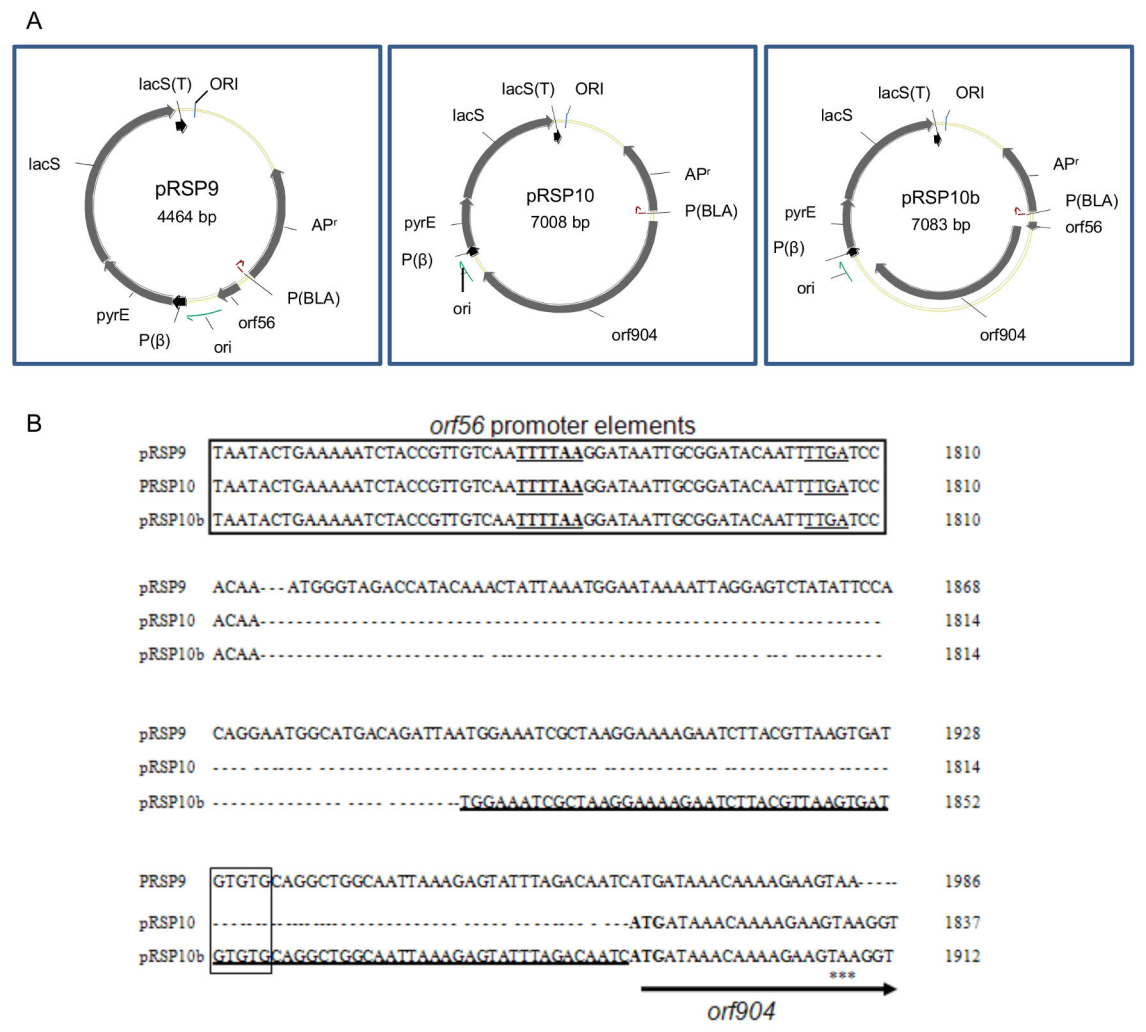
*In-trans* complementation of *orf56* and *orf904* deletion mutants of pRSP1. (A) Deletion mutants of pRSP1 generated by deleting *orf904* (pRSP9) and *orf56* (pRSP10/pRSP10b) from pRSP1; pRSP10 contained a complete *orf904* under the control of *orf56* transcription and translation elements, while pRSP10b contained an additional 75-bp at the 5’ of *orf904* start codon. Each plasmid contains pUC19 and pRN1 origins of replication, the *pyrE-lacS* cassette transcribed by the S. *solfataricus* *thsB* promoter [P(β)] and *lacS* terminator element [lacS(T)], and AP^r^ gene (ampicillin resistance). (B) *Cis*-acting elements at the 5’ of *orf56* and *orf904* in each plasmid. Transcription of *orf904* in pRSP10 and pRSP10b is controlled by of *orf56* promoter elements (big bold box) with putative TATA box A (bold single underline) and TATA box B (single underline). The 75-bp element 5’of *orf904* in pRSP10b (bold underline) contains a putative RBS (small box). The start of *orf904* is bolded, and the stop of *orf56* is represented by (***).

We attempted to verify the report that *orf56* and *orf904* are critical for pRN1 replication [7,25] by making in-frame deletions of each gene from pRSP1 generating pRSP10 and pRSP9, respectively (Figure 5). Each of the genes was placed under the control of the putative promoter elements of the co-transcribed genes [22] located at the 5’-end of *orf56*. We consistently recovered transformants with pRSP1 but not with pRSP9 or pRSP10 (Table 4), confirming the importance of these genes as previously reported [7,25]. We explored the possibility of complementing the genes *in-trans* by transforming both plasmids into *S. acidocaldarius E4.39*, but did not recover transformants with the pair of plasmids. We evaluated the possibility that some elements at the 5’ of *orf904* start codon might be important for transcription and translation of the gene by generating a third plasmid pRSP10b (Figure 5). The new plasmid (pRSP10b) differs from pRSP10 by the presence of the contiguous 75-bp sequence at the 5’ of *orf904* start codon (Figure 5B). As expected, transformation of pRSP10b into *S. acidocaldarius E4-39* did not produce transformants (Table 4). However, LacS-expressing transformants were recovered with the pRSP9/pRSP10b pair, albeit with a very low efficiency (Table 4). The pRSP9/pRSP10b transformants grew very poorly in liquid medium, making it challenging to recover plasmid DNA for further analysis. The reason for poor growth of the pRSP9/pRSP10b is not known but the transformation result was consistent after more than three attempts. In addition to the suggestion that some elements within the additional 75-bp sequence in pRSP10b might be important for plasmid function; the result from the pRSP9/pRSP10b transformation highlights the possibility of transforming *S. acidocaldarius* with more than one plasmid.

**Table 4:** Co-transformation of pRSP9 with pRSP10 or pRSP10b.

**Plasmid(s**)	**Deleted region**	**Length**	**Gene affected**	**Transformation yield (cfu/ng**)***^a^*,*^b^***
pRSP9	1986 - 4680	2695	orf904	Nil
pRSP10	1815 - 1965	151	orf56	Nil
pRSP10b	1815 - 1890	76	orf56	Nil
pRSP9 + pRSP10				Nil
pRSP9 + pRSP10b				8.2 × 10° – 4.9 × 10

^a^ The transformation experiments were carried out at least three times. The efficiency varied with experiments

^b^ Negative control (without plasmid DNA) and positive control (pRSP1 or pRSP3) reactions were always included with each set of experiments

## Discussion

We functionally characterized a 232-bp region in pRN1 located at the 3’ of *orf904* as the putative origin of replication of the plasmid [22,23,27]. The origin of replication of pRN1, which replicates by a rolling circle mechanism, has not been well characterized [25] despite its increased application as a vehicle for transformation of *Sulfolobus* species [7,20]. The 232-bp A-T rich stretch of DNA was identified by visual inspection of the entire sequence without the annotations and coincided with the region that was previously suggested to be part of the minimum replicon of the plasmid [25]. The function of the putative origin was characterized using pRSP1 – pRSP7, shuttle vectors harboring the 232-bp region and various lengths of pRN1. Each shuttle vector produced *S. acidocaldarius E4-39* transformants (Table 2 and Figure S3), indicating that it contained elements essential for plasmid replication. We deduced that the pRN1 fragment present in pRSP1 and pRSP2—which includes *orf56*, *orf904*, and the 232-bp region (Figure 1)—was sufficient for replication of pRN1. The potential role of the 232-bp region as the putative origin was further highlighted by the inability of mutants lacking this region (pRSP1-NO and pRSP3-NO) to produce transformants after numerous attempts (Table 3). We also demonstrated that deletion of *orf56* or *orf904* from pRSP1 completely eliminated the ability of the resulting plasmids (pRSP10 and pRSP9, respectively) to produce transformants (Table 4), confirming previous reports that the genes are essential for pRN1 replication [7,25]. Based on our results we concluded that the co-transcribed *orf56* and *orf904* in pRSP1 and the 232-bp region downstream constitute the minimum replication unit of pRN1 in agreement with previous reports [7,25]. 

The proposed 232-bp origin of replication of pRN1 appears to be conserved within the pRN plasmid family, especially with pHEN7 (Figure S5). The 232-bp element contains inverted repeats (Figure 4) that form stem-loop structures that are required for rolling circle replication [28,29]. Our results indicate that a proposed 100-bp stem-loop structure (Figure 4) within the 232-bp putative origin might function as the double-strand origin of replication [28]. The importance of this 100-bp region was highlighted by the inability of plasmids constructed by deleting 3 - 60 bp within this structure in pRSP1 to yield transformants (Figure 2) compared to the wild-type plasmid (Table 3). The IRII arm of the ‘cruciform’ structure that harbors the ‘nick site’ in double-strand origins of rolling circle plasmids is critical for plasmid function [30-32]. 

The loop of the 100-bp stem-loop structure harbors a G-C rich region that includes a GTG motif (Figure 4), a feature that has been reported to be essential for Orf904 primase activity [35]. The importance of the GTG motif and the loop of the 100-bp structure were highlighted by the inability of pRSP1 to tolerate deletion of the GTG motif or 3-bp from the adjacent regions, resulting in loss of plasmid transformation (Table 3). In comparison, deletion of 12-bp harboring another ‘GTG’ motif located at the 3’ of the 100-bp structure from pRSP1 (pRSP1e) did not impact the ability of the plasmid to replicate and produce transformants (Table 3). Based on these results and the critical role of the critical loop of the 100-bp stem-loop structure, we propose that the structure is the site of initiation of pRN1 replication. We believe that the 58-bp loop contains the ‘nick site’ for pRN1 replication. The 58-bp look was sensitive to deletion of 3 bp but insensitive to base-pair substitution, as few single base-pair substitutions (A → G) within the loop of the 100-bp stem-loop in pRSP1 impacted the ability of the resulting plasmids to produce transformants. We suspect that the ‘nick site’ for initiating pRN1 replication could be located a few base pairs 3’ of the GTG motif, but we have yet to identify it. 

The regions immediately 5’ and 3’ of the 100-bp stem-loop structure—which were deleted in pRSP1d and pRSP1a, respectively (Figures 2 and 4)—appeared to be important, as both plasmids yielded transformants that grew poorly on solid medium compared to those transformed with pRSP1 (Figure S4). In addition, the pRSP1d transformants failed to grow in liquid medium, while cells transformed with pRSP1a grew poorly in liquid medium. The reason for the poor growth of pRSP1a and pRSP1d transformants is not clear, but we suspect plasmid instability since pRSP1 transformants grew normally in liquid and solid media. Therefore, we hypothesize that the region 5’ of the 100-bp structure might function as a single-strand origin based on its location, while the 3’ region could potentially function in termination of plasmid replication [28-32]. However, further studies would need to be conducted to verify the role of the region up- and downstream of the 100-bp stem-loop. 

In line with our goal to develop tools for engineering *Sulfolobus*, we explored the possibility of transforming the organism with multiple plasmids and demonstrated that pRSP9 and pRSP10b can be co-transformed *in-trans*. Interestingly, we were unable to co-transformed pRSP9 and pRSP10, suggesting that there might be some important elements within the 75-bp sequence at the 5’-end of *orf904*. We expected to recover transformants with the pRSP9/pRSP10 pair, because the transcription and translation of *orf904* was directed by the *orf56* regulatory elements in pRSP10 (Figure 5 and Table 4). The inability to recover transformants with the pRSP9 – pRSP10 pair, suggests that the important element within the additional 75-bp in pRSP10b is/are *cis*-acting because the entire 75-bp sequence is also present in pRSP9. Although transformants were obtained from pRSP9/pRSP10b transformations, these transformants grew poorly in liquid medium, hampering our efforts to recover the plasmids for analysis. We have not elucidated the reason for poor growth of pRSP9/pRSP10b transformants in liquid medium, but we cannot rule out plasmid incompatibility owing that both plasmids have the same origin. There is also the possibility of some form of recombination between pRSP9 and pRSP10b or between the plasmids and the chromosome. The presence of transformants with the pRSP9/pRSP10b pair indicated that *orf56* and *orf904* were functionally expressed and possibly acted *in trans*, since both genes are essential for replication of the plasmid [7,25]. The nature or role of the element(s) in this 75-bp region is not known. We speculate that *orf904* was transcribed by the promoter of *orf56* and subsequently translated by a putative RBS in *orf56* (Figure 5). However, we are not ruling out the possibility that the 75-bp region 5’ of *orf904* contains *cis*-acting element(s) that might be essential for initiating transcription (Figure 5B). 

Transformation of *S. acidocaldarius* with pRSP9 and pRSP10b also highlighted the possibility of transforming *S. acidocaldarius* with multiple plasmids, even those with the same origin of replication. The poor growth of the double transformant suggests that there might be some plasmid compatibility issues; however, transformation with multiple plasmids expands the application of pRN1-based plasmids. Further work needs to be carried out to understand why the two-plasmid transformants grew poorly in effort to improve these tools. 

In summary, we have refined and clarified the functions of various elements in the origin of replication of pRN1, the cryptic plasmid from *S. islandicus REN1H1*. The proposed double-strand origin of the plasmid was identified as the most critical *cis*-acting element for replication of the plasmid. We also proposed a potential single-strand origin for the replication of the lagging strand, but we did not identify the ‘nick site’ within the loop of the double-strand origin. We also demonstrated that two pRN1-based shuttle vectors can be co-transformed in *S. acidocaldarius* and selected using a single auxotrophic marker by splitting the essential *trans*-acting Orf56 and Orf904. To enhance the utility of these shuttle vectors, we constructed two additional plasmids pRSP1-CL and pRSP2-CL by adding multiple cloning sites to pRSP1 and pRSP2, respectively (Figure S7). Further studies need to be performed to fully understand the detailed mechanism of replication of this plasmid.

## Supporting Information

Figure S1 **Selection of uracil auxotroph.** *S. acidocaldarius* was grown for 6 days on liquid YTFU medium (YT supplemented with 5 µg/ml uracil and 50 µg/ml 5-fluoroorotic acid [5-FOA]) at 75°C (200 rpm) to select spontaneous uracil auxotroph. Enriched uracil auxotrophic strains were sub-cultured in YTFU, YT and YTF (YT + 50 µg/ml 5-FOA) and growth was observed only in YTFU after 48 h incubation (A). The presence of uracil auxotrophs in YT and YTF culture tubes was confirmed by adding 20 µg/ml uracil to each tube and incubated for additional 48 h (B). Prior to selective enrichment of spontaneous uracil auxotrophic strains, lawns/colonies were observed on YT plates, but not on YTF or YTFU plates, but after enrichment, lawns/colonies were observed only on YTFU plates; a few revertants were observed on YT plate inoculated with un-diluted culture (C). Strain E4-39 with duplication of a 17-bp (blue and red) sequence within *pyrE* was selected for this study after characterization of the auxotrophic strains on YTFU plates (D). The *pyrEF* genes were amplified from the genomic DNA extracted from selected mutants (DNeasy blood and tissue kit, Qiagen) and sequenced to determine the mutations in mutants.
(TIF)

Figure S2 **Construction of pGlcSTUV_ko.** The plasmid was constructed for knocking out the Saci_1163 – Saci_1165 gene cluster, which is suspected to encode the putative glucose ABC transporter. The vector was constructed by inserting *S. solfataricus* (Sso) *pyrE-lacS* cassette into pUC19 backbone. The Sso *pyrE-lacS* cassette consists of *pyrE* and *lacS* artificial operon under the control of 282) thermosome B-subunit (*thsB* – Sso 0282) promoter element located 100 bp upstream of the Sso0282 start codon. Translation of *lacS* is translated by the putative RBS of *pyrF* and is kept in-frame by the addition of an “A-T” pair between the stop codon of *pyrE* and the start codon of *lacS*. The terminator element of *lacS* was added by including 80 bp downstream of its stop codon. Sso *pyrE* serves as a marker for blue-white screening.
(TIF)

Figure S3 **Transformation of pRSP1 – 7 into *S. acidocaldarius E4-39*.** The entire shuttle vectors harboring pRN1 fragments replicated in *S. acidocaldarius* *E4-39*. Cells transformed with pRSP1 (b), pRSP3 (d), pRSP5 (f) and pRSP7 (h) yielded blue colonies because of the presence of LacS, while pRSP2 (c), pRSP4 (e) and pRSP6 (g) transformants yielded white colonies because of the absence of LacS. Large colonies seen on all plates are revertants and are easily distinguished from the transformants, which are smaller. The cells were transformed by electroporation and no plasmid was added to the control sample (a).
(TIF)

Figure S4 **Analyzing pRN1 origin of replication.** Regions of pRN1 putative origins were deleted from pRSP1 and transformed into *S. acidocaldarius* *E4-39* (A). Plates show cells with pRSP1a (i), pRSP1b (ii), pRSP1c (iii), pRSP1d (iv), pRSP1e (v) and pRSP1 (vi). Inserts in (i) and (ii) highlight enlarged tiny blue colonies from pRSP1a and pRSP1d, respectively. The role of the pRN1 putative origin in pRSP3 replication was determined (B) by transforming the S. *acidocaldarius* *E4-39* with no plasmid (i), pRSP3 (ii) and pRSP3-NO which lacks the entire putative origin except 35 bp at the 5’ end (iii). Similar results were obtained with pRSP1-NO, which lacks the entire putative origin except 9 bp at the 5’-end (not shown).
(TIF)

Figure S5 **Alignment putative origin of replication of pRN1 with members of the pRN family of plasmid.** The putative origin of pRN1 aligned with regions 3’ of the *orf904* homologs in pRN2, pDL10, and pHEN7. The loop of the 100-bp stem-loop (underlined) is shaded gray. The consensus sequence with ‘GTG’ motif is highlighted (box). The putative origin region of pRN1 corresponds to the predicted single-strand origin of pHEN7 and pDL10.
(TIF)

Figure S6 **Secondary structure of the putative origin of replication at 37°C.** The mFold structure highlights potential hairpin structures at the 5’- and 3’-ends of the putative origin. The regions deleted in pRSP1a – pRSP1d are highlighted (arrows).
(TIF)

Figure S7 **Map of pRSP1-CL and pRSP2-CL.** Multiple cloning sites including of HindIII-XhoI-AatII-NruI-PacI-PstI-PvuII-NsiI (MCS) were added to pRSP1 (A) and pRSP2 (B) to generate pRSP1-CL (C) and pRSP2-CL (D) to enhance its utility for cloning.
(TIF)

Table S1 Description of primers used in plasmid construction
(DOCX)
