# Supplementary figures and images for: Functional Characterization of the Origin of Replication of pRN1 from Sulfolobus islandicus REN1H1
Source: PLoS One. 2013 Dec 20;8(12):e84664. doi: 10.1371/journal.pone.0084664 (PMC3869888; doi:10.1371/journal.pone.0084664)

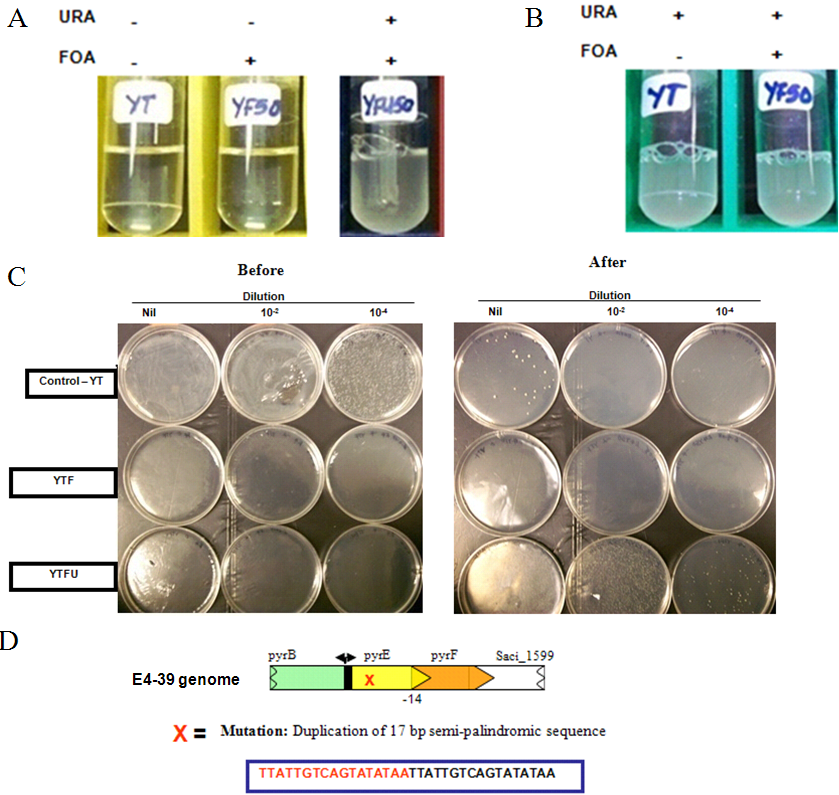

Supplement: Figure S1 — Selection of uracil auxotroph. S. acidocaldarius was grown for 6 days on liquid YTFU medium (YT supplemented with 5 µg/ml uracil and 50 µg/ml 5-fluoroorotic acid [5-FOA]) at 75°C (200 rpm) to select spontaneous uracil auxotroph. Enriched uracil auxotrophic strains were sub-cultured in YTFU, YT and YTF (YT + 50 µg/ml 5-FOA) and growth was observed only in YTFU after 48 h incubation (A). The presence of uracil auxotrophs in YT and YTF culture tubes was confirmed by adding 20 µg/ml uracil to each tube and incubated for additional 48 h (B). Prior to selective enrichment of spontaneous uracil auxotrophic strains, lawns/colonies were observed on YT plates, but not on YTF or YTFU plates, but after enrichment, lawns/colonies were observed only on YTFU plates; a few revertants were observed on YT plate inoculated with un-diluted culture (C). Strain E4-39 with duplication of a 17-bp (blue and red) sequence within pyrE was selected for this study after characterization of the auxotrophic strains on YTFU plates (D). The pyrEF genes were amplified from the genomic DNA extracted from selected mutants (DNeasy blood and tissue kit, Qiagen) and sequenced to determine the mutations in mutants. (TIF) [file pone.0084664.s001.tif]

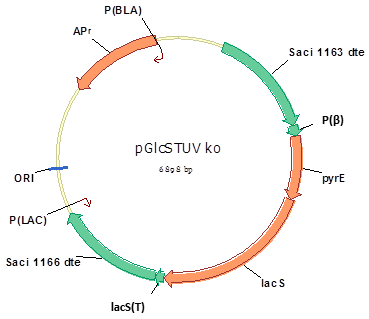

Supplement: Figure S2 — Construction of pGlcSTUV_ko. The plasmid was constructed for knocking out the Saci_1163 – Saci_1165 gene cluster, which is suspected to encode the putative glucose ABC transporter. The vector was constructed by inserting S. solfataricus (Sso) pyrE-lacS cassette into pUC19 backbone. The Sso pyrE-lacS cassette consists of pyrE and lacS artificial operon under the control of 282) thermosome B-subunit (thsB – Sso 0282) promoter element located 100 bp upstream of the Sso0282 start codon. Translation of lacS is translated by the putative RBS of pyrF and is kept in-frame by the addition of an “A-T” pair between the stop codon of pyrE and the start codon of lacS. The terminator element of lacS was added by including 80 bp downstream of its stop codon. Sso pyrE serves as a marker for blue-white screening. (TIF) [file pone.0084664.s002.tif]

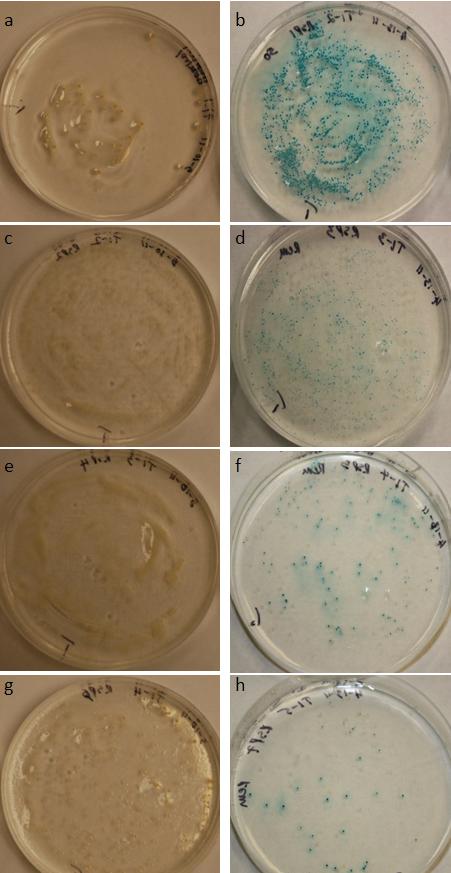

Supplement: Figure S3 — Transformation of pRSP1 – 7 into S. acidocaldarius E4-39. The entire shuttle vectors harboring pRN1 fragments replicated in S. acidocaldarius E4-39. Cells transformed with pRSP1 (b), pRSP3 (d), pRSP5 (f) and pRSP7 (h) yielded blue colonies because of the presence of LacS, while pRSP2 (c), pRSP4 (e) and pRSP6 (g) transformants yielded white colonies because of the absence of LacS. Large colonies seen on all plates are revertants and are easily distinguished from the transformants, which are smaller. The cells were transformed by electroporation and no plasmid was added to the control sample (a). (TIF) [file pone.0084664.s003.tif]

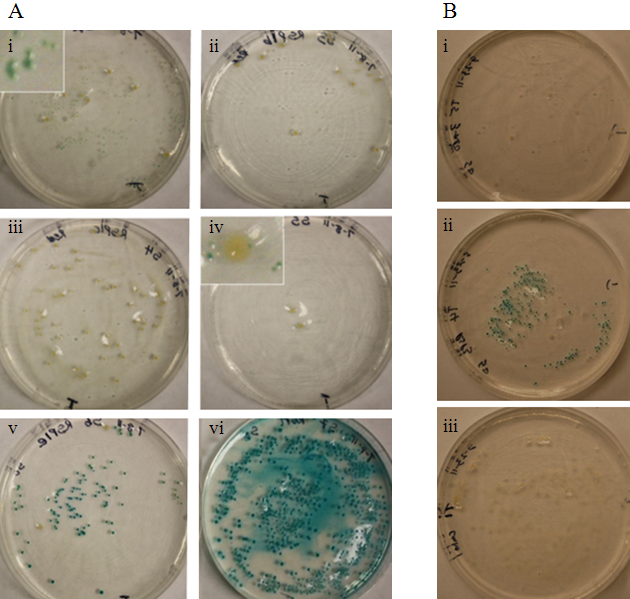

Supplement: Figure S4 — Analyzing pRN1 origin of replication. Regions of pRN1 putative origins were deleted from pRSP1 and transformed into S. acidocaldarius E4-39 (A). Plates show cells with pRSP1a (i), pRSP1b (ii), pRSP1c (iii), pRSP1d (iv), pRSP1e (v) and pRSP1 (vi). Inserts in (i) and (ii) highlight enlarged tiny blue colonies from pRSP1a and pRSP1d, respectively. The role of the pRN1 putative origin in pRSP3 replication was determined (B) by transforming the S. acidocaldarius E4-39 with no plasmid (i), pRSP3 (ii) and pRSP3-NO which lacks the entire putative origin except 35 bp at the 5’ end (iii). Similar results were obtained with pRSP1-NO, which lacks the entire putative origin except 9 bp at the 5’-end (not shown). (TIF) [file pone.0084664.s004.tif]

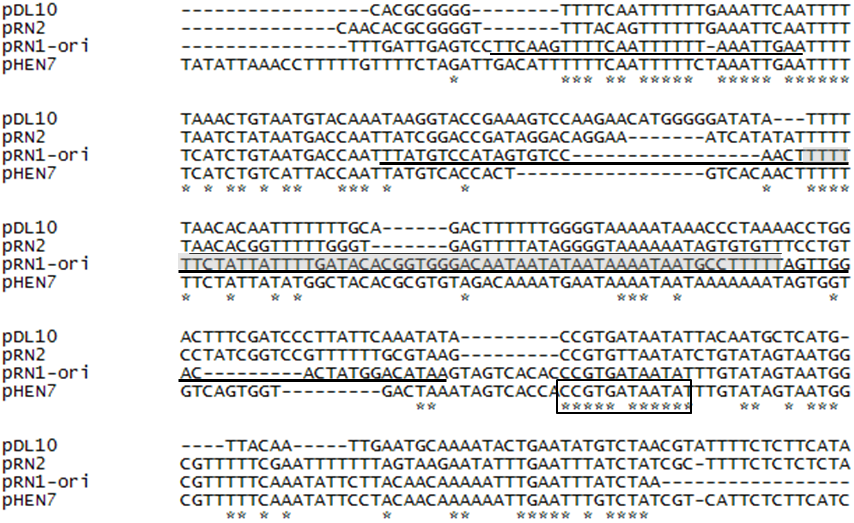

Supplement: Figure S5 — Alignment putative origin of replication of pRN1 with members of the pRN family of plasmid. The putative origin of pRN1 aligned with regions 3’ of the orf904 homologs in pRN2, pDL10, and pHEN7. The loop of the 100-bp stem-loop (underlined) is shaded gray. The consensus sequence with ‘GTG’ motif is highlighted (box). The putative origin region of pRN1 corresponds to the predicted single-strand origin of pHEN7 and pDL10. (TIF) [file pone.0084664.s005.tif]

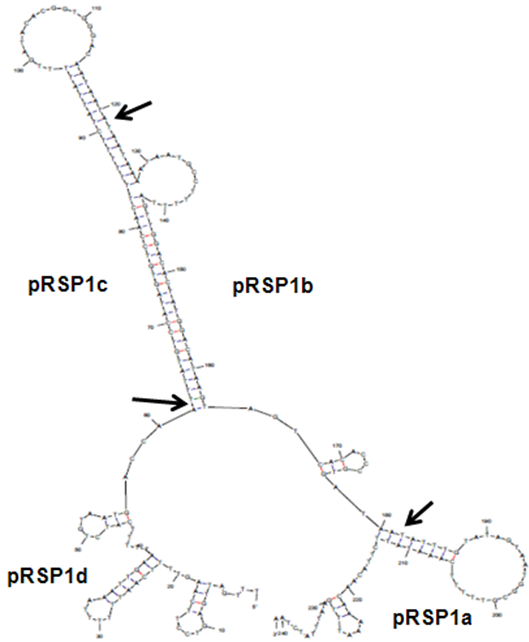

Supplement: Figure S6 — Secondary structure of the putative origin of replication at 37°C. The mFold structure highlights potential hairpin structures at the 5’- and 3’-ends of the putative origin. The regions deleted in pRSP1a – pRSP1d are highlighted (arrows). (TIF) [file pone.0084664.s006.tif]

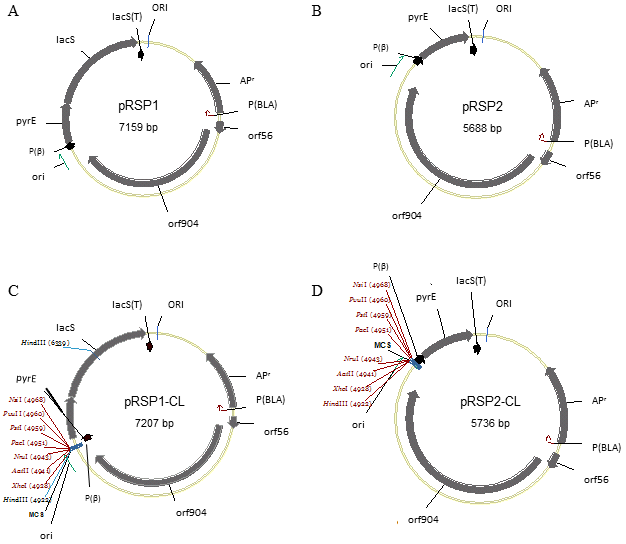

Supplement: Figure S7 — Map of pRSP1-CL and pRSP2-CL. Multiple cloning sites including of HindIII-XhoI-AatII-NruI-PacI-PstI-PvuII-NsiI (MCS) were added to pRSP1 (A) and pRSP2 (B) to generate pRSP1-CL (C) and pRSP2-CL (D) to enhance its utility for cloning. (TIF) [file pone.0084664.s007.tif]
