# Supplementary material for: Functional Characterization of the Origin of Replication of pRN1 from Sulfolobus islandicus REN1H1
Source: PLoS One. 2013 Dec 20;8(12):e84664. doi: 10.1371/journal.pone.0084664 (PMC3869888; doi:10.1371/journal.pone.0084664)
Supplement: Table S1 — Description of primers used in plasmid construction (DOCX) [file pone.0084664.s008.docx]

**Table S1.** Primers

| **P/N** | **Name** | **Length (bp)** | | **Amplicon** | **Sequence** |
| --- | --- | --- | --- | --- | --- |
| **117** | stuv-ss-lacS-RT | 20 | | pyrE-lacS fragment | AAAGTATTGCAATCTAATGA |
| **120** | stuv-ss-tf55b-upe-F | 18 | | pyrE-lacS fragment | AAACGCTTAACATTACTT |
| **128** | RUC-F | 50 | | pUC19 fragment | TCTAATCTCATTTTCATTAGATTGCAATACTTTAAAGAACATGTGAGCAA |
| **129** | RUC-R | 50 | | pUC19 fragment | AAATTGACAACGGTAGATTTTTCAGTATTATATGTATCCGCTCATGAGAC |
| **130** | RC-F | 18 | | pRSP1, 3, 5 and 7 fragments | TAATACTGAAAAATCTAC |
| **131** | RC-R1 | 50 | | pRSP1 fragment | AAGTTTATCGAAAAGTAATGTTAAGCGTTTGAATGACGTTAGATAAATTC |
| **132** | RC-R3 | 50 | | pRSP3 fragment | AAGTTTATCGAAAAGTAATGTTAAGCGTTTTTAATCATATCTGTGATTGT |
| **133** | RC-R5 | 48 | | RSP5 fragment | AAGTTTATCGAAAAGTAATGTTAAGCGTTTGTATCATTATGTATCGAT |
| **134** | RC-R7 | 50 | | RSP7 fragment | AAGTTTATCGAAAAGTAATGTTAAGCGTTTCGGCATAAGTAGGAATAGTT |
| **135** | RS-E-F | 50 | | pRSP2, 4 and 6 | GATTATTTGGTGAAGAATGTTGAAAAGTAGACTTTCTCAAGTCTCACTAT |
| **136** | RS-E-R | 20 | | pRSP2, 4 and 6 | CTACTTTTCAACATTCTTCA |
| **137** | pCN-F | 50 | | pRSP3-NO | TTGAGTCCTTCAAGTTTTCAATTTTTTAAACGTCATTCTCTCTATAGAAC |
| **138** | pCN-R | 28 | | pRSP3-NO | TTTAAAAAATTGAAAACTTGAAGGACTC |
| **160** | RSP9-F | 17 | | pRSP9 | GTCCTTCAAGTTTTCAA |
| **161** | RSP9-R | 49 | | pRSP9 | TCAATTTAAAAAATTGAAAACTTGAAGGACTTACTTCTTTTGTTTATCA |
| **162** | RSP10-F | 50 | | pRSP10 | GATAATTGCGGATACAATTTTGATCCACAAATGATAAACAAAAGAAGTAA |
| **165** | RSP1-CLF | 58 | | pRSP1-CL/pRSP2-CL | AGTTTGACGTCGCGATTAATTAACTGCAGCTGATGCATAAACGCTTAACATTACTTTT |
| **166** | RSP1-CLR | 60 | | pRSP1-CL/pRSP2-CL | GCTGCAGTTAATTAATCGCGACGTCAAACTCGAGAAGCTTGAATGACGTTAGATAAATTC |
| **167** | RSP1a-F | 50 | | pRSP1a | TATGGACATAAGTAGTCACACCCGTGATAACGTCATTCAAACGCTTAACA |
| **168** | RSP1a-R | 20 | | pRSP1a | TTATCACGGGTGTGACTACT |
| **169** | RSP1b-F | 50 | | pRSP1b | ATTATTTTGATACACGGTGGGACAATAATATATTTGTATAGTAATGGCGT |
| **171** | RSP1c-F | 50 | | pRSP1c | TAAATTGAATTTTTCATCTGTAATGACCAATAATAAAATAATGCCTTTTT |
| **172** | RSP1c-R | 18 | | pRSP1c | TTGGTCATTACAGATGAA |
| **173** | RSP1d-F | 50 | | pRSP1d | GATTGATGAAGAGCACGTGCGGTTTGATTGATTTATGTCCATAGTGTCCA |
| **179** | RSP10-R2 | 20 | | pRSP10 | GGATCAAAATTGTATCCGCA |
| **181** | RSP1e-R | 20 | | pRSP1e | GTGTGACTACTTATGTCCAT |
| **182** | RSP10-F2 | 20 | | pRSP10 | ATGATAAACAAAAGAAGTAA |
| **184** | RSP1-CLF2 | 20 | | pRSP1-CL/pRSP2-CL | AAACGCTTAACATTACTTTT |
| **186** | RSP1a-F2 | 20 | | pRSP1a/pRSP1-NO | CGTCATTCAAACGCTTAACA |
| **187** | RSP1c-F2 | 20 | | pRSP1c | TAATAAAATAATGCCTTTTT |
| **189** | RSP1e-F2 | 18 | | pRSP1e | TTGTATAGTAATGGCGTT |
| **190** | RSP1-NO-R | 21 | | pRSP1-NO | TCAATCAAACCGCACGTGCTC |
| **191** | RSP1-L1F | 22 | | pRSP1-L1/pRSP1-L4 | AGTTGGACACTATGGACATAAG |
| **192** | RSP1-L1R | 22 | pRSP1-L1 | | TATTATTGTCCCACCGTGTATC |
| **193** | RSP1-L2F | 25 | pRSP1-L2 | | TAATAAAATAATGCCTTTTTAGTTG |
| **194** | RSP1-L2R | 23 | pRSP1-L2 | | TCAAAATAATAGAAAAAAAGTTG |
| **195** | RSP1-L3F | 20 | pRSP1-L3 | | TACACGGTGGGACAATAATA |
| **196** | RSP1-L3R | 18 | pRSP1-L3/pRSP1-L4 | | AGTTGGACACTATGGACA |
| **197** | RSP1-A1-F | 15 | pRSP1-A1 | | GGCAATAATATAATA |
| **198** | RSP1-A2-F | 15 | pRSP1-A2 | | GACGATAATATAATA |
| **199** | RSP1-A3-F | 15 | pRSP1-A3 | | GACAGTAATATAATA |
| **203** | RSP1-A7-F | 15 | pRSP1-A7 | | GACAATAATATGATA |
| **205** | RSP1-A1-8-R | 15 | pRSP1-A1 - pRSP1-A7 | | CCACCGTGTATCAAA |
| **206** | RSP1-G1-R | 23 | pRSP1-G1 and 2 | | TCAAAATAATAGAAAAAAAGTTG |
| **207** | RSP1-G1-F | 18 | pRSP1-G1 | | ACGGTGGGACAATAATAT |
| **208** | RSP1-G2-F | 21 | pRSP1-G2 | | TACGTGGGACAATAATATAAT |
| **209** | RSP1-G3-R | 18 | pRSP1-G3 and 4 | | CGTGTATCAAAATAATAG |
| **210** | RSP1-G3-F | 21 | pRSP1-G3 | | GGACAATAATATAATAAAATA |
| **211** | RSP1-G4-F | 24 | pRSP1-G4 | | GTGCAATAATATAATAAAATAATG |
| **212** | RSP1-G5-R | 17 | pRSP1-G5 and 6 | | TCCCACCGTGTATCAAA |
| **213** | RSP1-G5-F | 20 | pRSP1-G5 | | TAATATAATAAAATAATGCC |
| **214** | RSP1-G6-F | 22 | pRSP1-G6 | | CAATATAATAAAATAATGCCTT |
| **215** | RSP1-G7-R | 20 | pRSP1-G7 and 8 | | TTATTGTCCCACCGTGTATC |
| **216** | RSP1-G7-F | 24 | pRSP1-G7 | | AATAAAATAATGCCTTTTTAGTTG |
| **217** | RSP1-G8-F | 24 | pRSP1-G8 | | TATAAAATAATGCCTTTTTAGTTG |

Note: (1) Primer name ending in “F” indicates a forward primer and those ending in “R” indicate a reverse primer; (2) Underlined sequence in a primer indicates the annealing sequence, the extra bases at the 5’ are usually adaptors added to link the amplicon to adjacent fragments during plasmid assembly
